# Molecular Targets and Mechanisms of 6,7-Dihydroxy-2,4-dimethoxyphenanthrene from Chinese Yam Modulating NF-κB/COX-2 Signaling Pathway: The Application of Molecular Docking and Gene Silencing

**DOI:** 10.3390/nu15040883

**Published:** 2023-02-09

**Authors:** Congyi Nie, Yuxiao Zou, Sentai Liao, Qunyu Gao, Qian Li

**Affiliations:** 1 Guangdong Academy of Agricultural Sciences, Sericultural & Agri-Food Research Institute/Key Laboratory of Functional Foods, Ministry of Agriculture and Rural Affairs/Guangdong Key Laboratory of Agricultural Products Processing, Guangzhou 510610, China; 2 School of Food Science and Engineering, South China University of Technology, Guangzhou 510640, China

**Keywords:** Chinese yam polyphenols, phenanthrene, NF-κB/COX-2 signaling pathway, molecular docking, gene silencing

## Abstract

Chinese yam (*Dioscorea opposita*) tuber has a significant effect of invigorating the intestine and improving the symptoms of long-term diarrhea according to the records of the Chinese Pharmacopoeia. Phenanthrene polyphenols from Chinese yam, with higher inhibition of cyclooxygenase-2 (COX-2) than anti-inflammatory drugs, are an important material basis in alleviating ulcerative colitis via nuclear factor kappa-B (NF-κB)/COX-2 pathway, based on our previous research. The present study further explored the target and molecular mechanisms of phenanthrenes’ modulation of the NF-κB/COX-2 signaling pathway by means of molecular docking and gene silencing. Firstly, interleukin-8 (IL-8) and tumor necrosis factor-α (TNF-α) expression of 6-hydroxy-2,4,7-trimethoxyphenanthrene (PC2)/6,7-dihydroxy-2,4-dimethoxyphe-nanthrene (PC4) were compared on TNF-α induced human colon adenocarcinoma (Caco-2) cells. Secondly, molecular docking and dynamics simulation were implemented for PC2/PC4 and COX-2. Finally, COX-2 silencing was performed on TNF-α induced Caco-2 cells to confirm the target of PC4 on NF-κB/COX-2 pathway. Lower expression of IL-8 and TNF-α in PC4 treated Caco-2 cells indicated that PC4 had stronger anti-inflammatory activity than PC2. The binding of PC4 and COX-2 was stronger due to the hydrogen bond between hydroxyl group and Tyr385. No significant differences were found in phosphorylation nuclear factor kappa-B inhibitor alpha (pIkBα), phosphorylation NF-κB (pNF-κB) and phosphorylation extracellular signal-regulated kinase 1/2 (pERK1/2) expression between control and PC4 group after silencing, while these protein expressions significantly decreased in PC4 group without silencing, which confirmed that COX-2 was the important target for PC4 in alleviating ulcerative colitis. These findings indicate that PC4 was supposed to have inhibited NF-κB pathway mediated inflammation via suppression of positive feedback targeting COX-2.

## 1. Introduction

Chinese Yam (*Dioscorea opposita*) tubers have been consumed for more than 3000 years [1]. They contain many nutrients such as starch, protein, free amino acids and vitamins, and are rich in bioactive substances including polyphenols, polysaccharides and saponins [2,3]. Polyphenols in yam display antioxidant, anti-neuroinflammatory and nitrite elimination activities. Woo et al. demonstrated that phenolic derivatives obtained from ethanol extract of *Dioscorea nipponica* possessed good neuroprotective activity in C6 glioma cells [4]. Yam polyphenols were also found to inhibit the production of nitric oxide (NO) in inflammatory cells [5] and selectively reduce cyclooxygenase-2 (COX-2) activity, thus suggesting their anti-inflammatory effect [6]. In recent years, phenanthrene polyphenols extracted from plants or synthetic phenanthrene compounds have attracted wide attention due to their good anti-inflammatory, antioxidant and antibacterial effects [7], much preliminary research focusing on the anti-inflammatory activity of phenanthrene compounds in vitro [8,9,10]. However, few reports have explored the in-depth anti-inflammatory mechanism clarification of Chinese yam polyphenols, especially the phenanthrene polyphenols.

The cell inflammation model is widely used in vitro to analyze the bioactivity of phytochemicals. Abbasi et al. compared the cellular inflammation inhibitory effects of polyphenols obtained from agro-industrial by-products and observed that the expression of inflammatory factor interleukin-8 (IL-8) in human colon adenocarcinoma (Caco-2) cells was greatly reduced with grape pomace and spent coffee ground polyphenol extracts treatment [11]. Red cardoon stalk polyphenols inhibited lipopolysaccharide-induced inflammation by resisting the secretion of IL-8, IL-6, tumor necrosis factor-α (TNF-α) and IL-10 in Caco-2 cells [12]. In addition, phenolic compounds in navy bean and light red kidney bean milks have strong anti-inflammatory effects in a Caco-2/EA.hy926 co-culture model and these phenols were verified to regulate oxidized low density lipoprotein-induced inflammatory mediators through the mitogen-activated protein kinase (MAPK) pathway [13].

Nuclear factor kappa-B (NF-κB) plays a key role in inflammatory response, entering the nucleus to transcribe various proinflammatory factors [14]. The activation of NF-κB during inflammatory response is regulated by different kinases such as extracellular signal-regulated kinase, p38 protein kinase and c-Jun N-terminal kinase/stress activated protein kinase [15,16,17]. COX-2 enzyme is mainly located in the nuclear membrane and regulated by NF-κB. When tissue is stimulated by inducers, phospholipase A2 is activated to hydrolyze cell membrane phospholipids into arachidonic acids, which are catalyzed by COX-2 enzymes to produce prostaglandins (PGs). It has been proved that PGs exist in the positive feedback loop of chronic diseases [18]. Chronic mastitis response was induced by a COX-2-prostaglandin E2 (PGE2)-E-series of prostaglandin receptors type 2 (EP2)-NF-κB positive feedback loop, which suggested a potential mechanism of anti-inflammatory activity after COX-2 was inhibited as a drug target. Besides, bioactive substances could inhibit inflammation by down-regulating the expression of NF-κB, inhibiting COX-2 enzymes and inactivating PGs involved in the inflammatory process [19]. Glycytol was observed to inhibit the NF-κB pathway in lipopolysaccharide-induced macrophages and then down-regulated the expression of COX-2 protein [20]. At present, there are few studies focusing on research into the target role of COX-2 and the molecular mechanism.

**Figure 1:**
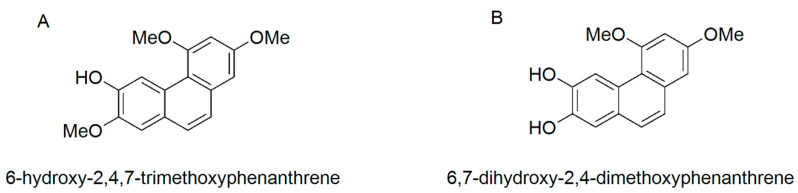
Molecular structure of (**A**) 6-hydroxy-2,4,7-trimethoxyphenanthrene (PC2) and (**B**) 6,7-dihydroxy-2,4-dimethoxyphenanthrene (PC4).

In our previous study, 6-hydroxy-2,4,7-trimethoxyphenanthrene (PC2, Figure 1A) and 6,7-dihydroxy-2,4-dimethoxyphenanthrene (PC4, Figure 1B) were isolated from the ethyl acetate extract of Chinese yam peel, which exhibited higher COX-2 inhibition than anti-inflammatory drugs such as aspirin, ibuprofen and naproxen [21]. PC4 alleviated dextran sulfate sodium salt (DSS)-induced intestinal mucosal injury in mice via modulation of the NF-κB/COX-2 pathway [22]. To further explore the target and molecular mechanism of the polyphenols in alleviating intestinal mucosal injury, the present study intended to compare the anti-inflammatory activities of PC2 and PC4 with a Caco-2 cell model and analyze the molecular docking and dynamics simulation between the two polyphenols and COX-2. Based on the Caco-2 inflammatory cell model, COX-2 gene silencing and western blot analysis, we revealed the target and molecular mechanism of PC4 in regulating the NF-κB/COX-2 signaling pathway. The research results will lay a theoretical foundation for the development of phytochemicals that alleviate ulcerative colitis.

## 2. Materials and Methods

### 2.1. Materials

Chinese yam characteristic polyphenols PC2 and PC4 were obtained from the ethyl acetate crude extract of Chinese yam polyphenol prepared by our research group. Colon adenocarcinoma (Caco-2) cells were derived from the American model culture collection (ATCC) and the cell generation is 30–50. TNF-α and IL-8 were obtained from Jiancheng Biological Engineering Institute, Nanjing, China, and 0.25% trypsin, Trizol reagent, reverse transcription primer (1 uM), moloney murine leukemia virus reverse transcriptase (200 U/μL), 2× PCR Master Mix, upstream primer (20 uM), downstream primer (20 uM), cDNA template, Taq DNA polymerase (2.5 U/μL), nuclear factor kappa-B inhibitor alpha (IkBα), phosphorylation IkBα (pIkBα), NF-κB, phosphorylation NF-κB (pNF-κB), extracellular signal-regulated kinase 1/2 (ERK1/2), phosphorylation ERK1/2 (pERK1/2) were supplied by Shanghai Shenggong, Shanghai, China. Acrylamide and N,N′-methylene bisacryl-amide were purchased from Sigma-Aldrich Trading Co., Ltd., Shanghai, China. Coomassie brilliant blue R250, Millipore Bløk™ Noise Cancelling Reagents chromogenic solution (A and B) were purchased from Shanghai Hengchuang Biotechnology Co., Ltd., Shanghai, China. All other reagents were of analytical grade.

### 2.2. Anti-Inflammatory Activity Test for PC2 and PC4

The Caco-2 cells were cultured in a CO_2_ incubator (Thermo Fisher Technology Co., Ltd., Waltham, MA, USA) according to our previous established method [23]. Then, 1 mL of Caco-2 cell suspension was seeded in 12-well plates at the concentration of 5 × 10^5^ cells/mL. The culture medium was changed every other day after cell adhesion and the culture medium was removed after 5–7 days. Subsequently, cells were rinsed 2–3 times with Hanks balanced salt solutions. The blank group consisted of cells without TNF-α addition. After intervention of PC2 and PC4 at 0.78, 1.56 and 3.125 μg/mL for 4 h, the model group and sample group were induced with TNF-α and the final concentrations were 100 ng/mL. The cell culture medium was collected after 4 h treatment and the levels of inflammatory factors TNF-α and IL-8 were detected according to kit methods.

### 2.3. Molecular Docking

Receptor preparation: High-resolution structure of COX-2 was downloaded from the Protein Data Bank with PDB-ID 3NT1 (www.rcsb.org, accessed on 4 March 2022). The pH value for COX-2 was set at 7.4. The protein optimization was completed by the Protein preparation Wizard program in Schrodinger software under the OPLS-2005 force field.

Ligand preparation: The 2D structures of PC2 and PC4 were drawn through ChemBioDraw 3D, then their mol2 format was outputted. LigPrep module was used for ligand preparation, pH value was set to 7.0 ± 2.0, tautomer state was determined by Epik program and other parameters were set by default. The Macromodel program was used to optimize molecules under the OPLS-2005 force field.

All docking studies were performed using the Schrodinger glide 2015-2 software. The docking region was determined by Receptor Grid Generation. The grid was set as default size and the van der Waals radius of proteins with partial atomic charge less than or equal to 0.25 was scaled by 1.0. Using the Glide XP docking scheme, PC2 and PC4 ligands were successively docked into the COX-2 protein active pocket, respectively. The binding place on the receptor was fixed before docking. COX-2 complex was downloaded from the Protein Data Bank with PDB-ID 5IKT (www.rcsb.org, accessed on 4 March 2022).

### 2.4. Molecular Dynamics Simulation

Molecular Dynamics simulations were carried out for COX-2 in complex with the PC2 and PC4. Each system was solvated in a cubic box with explicit TIP3P water and nutrient ions consisting of a solvent buffer region from the 10 Å edge of the complex. A 50 ns simulation was performed for the docked model by Amber 18 software under the isobaric-isothermal (NPT) condition at 298.15 K. The stability of the simulation was evaluated by root mean square deviation (RMSD). Besides, the binding free energy between proteins and ligands in all systems was calculated by the molecular mechanics generalized Born surface area (MM/PBSA) method [24].

### 2.5. Drug Intervention on Caco-2 Cells and Gene Silenced Caco-2 Cells

The Caco-2 cells were cultured according to our previous established method [22]. The cells were transfected with chemically synthesized COX-2 siRNA. Transfected cells were mixed with 500 μL serum-free medium at room temperature for 15–20 min. Then the cells were spread into a 6-well plate and the total volume of serum-free medium in each well was 2 mL. Blank normal Caco-2 cells (NC) and COX-2 silenced Caco-2 cells (Sh) were induced with 100 ng/mL TNF-α for 4 h, respectively. Phenanthrene polyphenol with better anti-inflammatory effect and more stable molecular combination was selected to treat cells. After the establishment of cellular inflammation model using TNF-α, blank group with TNF-α (BT), normal cell group with TNF-α (NT), COX-2 silenced cell group with TNF-α (sT), normal cell group with TNF-α and 0.78 μg/mL drug intervention (NTP0.78), normal cell group with TNF-α and 1.56 μg/mL drug intervention (NTP1.56), COX-2 silenced cell group with TNF-α and 0.78 μg/mL drug intervention (sTP0.78), COX-2 silenced cell group with TNF-α and 1.56 μg/mL drug intervention (sTP1.56), each group was treated with the drug for 24 h. Three groups of plasmid sequences (5′ to 3′) that transfected and silenced COX-2 gene were as follows:

siRNA-1 (CCCGGACAGGAUUCUAUGGAGAAAA),

siRNA-2 (GAAUAACAUUCCCUUCCUUCGAAAU),

siRNA-3 (CCAAAAUCGUAUUGCUGCUGAAUUU).

### 2.6. Western Blot Analysis

The expression levels of IkBα, pIkBα, NF-κB, pNF-κB, ERK1/2 and pERK1/2 proteins in Caco-2 cells were compared. The cell proteins were extracted with radio immunoprecipitation assay buffer, the protein concentration was determined with bicinchoninic acid assay kits, and 20 μg of the protein samples were separated by sodium dodecyl sulfate polyacrylamide gel electrophoresis in the Bio-Rad electrophoresis apparatus (USA Bio-Rad Company, Hercules, CA, USA) and then transferred to polyvinylidene fluoride (Millipore, Billerica, MA, USA) membranes. Electrophoretic proteins were sealed with 5% skimmed milk at room temperature for 2 h and membranes were incubated with primary antibody at 4 °C overnight. After washing the membrane with phosphate buffered saline with tween-20, the membranes were incubated with an appropriate amount of secondary antibody at room temperature for 2 h.

### 2.7. Statistical Analysis

Data were analyzed by SPSS software. One-way analysis of variance (ANOVA), non-parametric test and Welch test was used to determine significant differences between multiple groups. All data were expressed as mean ± standard deviation. The pairwise comparisons between groups were tested by LSD test and Tamhane T2 test and *p* < 0.05 was set as statistical significance.

## 3. Results

### 3.1. Comparison of PC2 and PC4 in Anti-Inflammatory Activities

**Figure 2:**
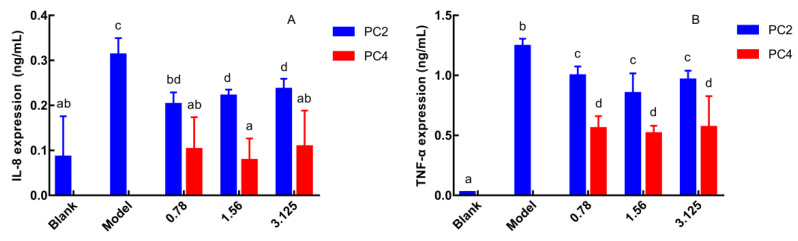
Effects of 6-hydroxy-2,4,7-trimethoxyphenanthrene (PC2) and 6,7-dihydroxy-2,4-di methoxy-phenanthrene (PC4) on the expression of inflammatory factors (**A**) Expression of interleukin-8 (IL-8), (**B**) Expression of tumor necrosis factor-α (TNF-α). Values without a common letter were significantly different as *p* < 0.05.

As pro-inflammatory cytokines, the expression level of IL-8 and TNF-α reflect severity of inflammation, and IL-8 is the strongest chemokine [25]. Therefore, the anti-inflammatory activities of phenanthrene polyphenols PC2 and PC4 were evaluated by the expression of IL-8 and TNF-α in Caco-2 cell culture supernatant. IL-8 and TNF-α expressions were the lowest in the blank group and the highest in the model group (Figure 2A,B). IL-8 and TNF-α expression in PC2 and PC4 groups were significantly reduced compared to the model group without a dose-dependent manner, which exhibited anti-inflammatory effects. Furthermore, both the IL-8 and TNF-α levels in PC4 group were significantly lower than in PC2, indicating that PC4 obtained the dominant ability in reducing the IL-8 and TNF-α expression. In general, PC4 possessed stronger anti-inflammatory activity than PC2, which was consistent with our COX-2 inhibition in vitro experiment [21].

### 3.2. Molecular Docking

#### 3.2.1. Molecular Docking Analysis

To further explore the anti-inflammatory mechanism and target of PC2 and PC4, g-score results and docking effect of ligand in complex with COX-2 protein were analyzed (Table 1A). The g-score is negative in glide docking and its greater absolute value indicates stronger binding force and better docking effect between ligand and receptor [26]. The absolute g-score value of PC4 (−8.109 kJ/mol) was greater than that of PC2 (−7.997 kJ/mol), showing that the docking effect of PC4 with COX-2 protein was better than PC2. The results explained why PC4 had stronger anti-inflammatory activity, which was consistent with anti-inflammatory activity results of two compounds in Caco-2 cells and COX-2 inhibition in in vitro experiment.

**Figure 3:**
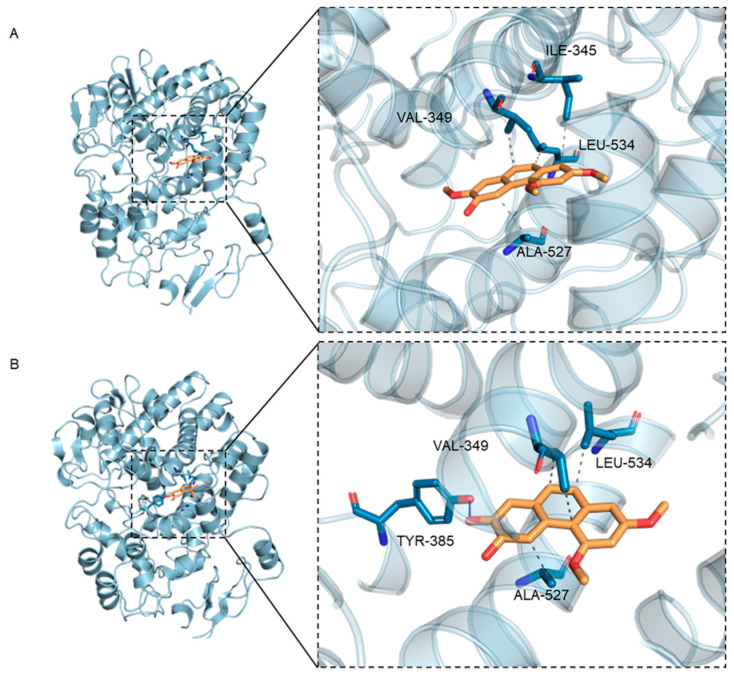
3D binding diagram of protein with different ligands (**A**) Binding of hydroxy-2,4,7-trimethoxyphenanthrene (PC2) to cyclooxygenase-2 (COX-2) protein, (**B**) Binding of 6,7-dihydroxy-2,4- dimethoxy-phenanthrene (PC4) to COX-2 protein. The left figure is the overall view and the right figure is the partial view. In the figure, yellow stick is small molecule and light blue cartoon is protein. The blue line indicates hydrogen bonding and the gray dotted line indicates hydrophobicity.

As shown in Table 1A and Figure 3, PC2 and PC4 occupied the active pocket formed by residues Ile345, Val349, Ala527, Leu534 and Tyr385 when docking with the COX-2 receptor protein. The docking analysis showed that PC4 bounded to COX-2 through one hydrogen bond and three hydrophobic interactions, while PC2 combined with protein through four hydrophobic interactions (Table 1A). Meanwhile, as a result of similar molecular structures, PC2 and PC4 had three identical amino acids including Val349, Ala527 and Leu534 at the binding sites in hydrophobic interaction. In addition, the 7′ carbon atom of PC2 and PC4 were connected with the methoxyl group and the hydroxyl group, respectively, resulting in different hydrogen bonds in the two compounds. The hydrogen bond between PC4 and COX-2 was formed by hydroxyl oxygen in polyphenols and hydroxyl group in Tyr385, while no hydrogen bond had been observed in complex of PC2 and protein, which might be the reason for the stronger binding between PC4 and COX-2.

#### 3.2.2. Molecular Dynamics Simulation Analysis

Biological entities have dynamic behavior under physiological conditions. However, the molecular docking results only provide the static state of the enzyme-inhibitor complexes. Therefore, we performed molecular dynamics simulation to study the dynamic movement of COX-2 active site during inhibition and herein reported the role of COX-2 active residues.

**Figure 4:**
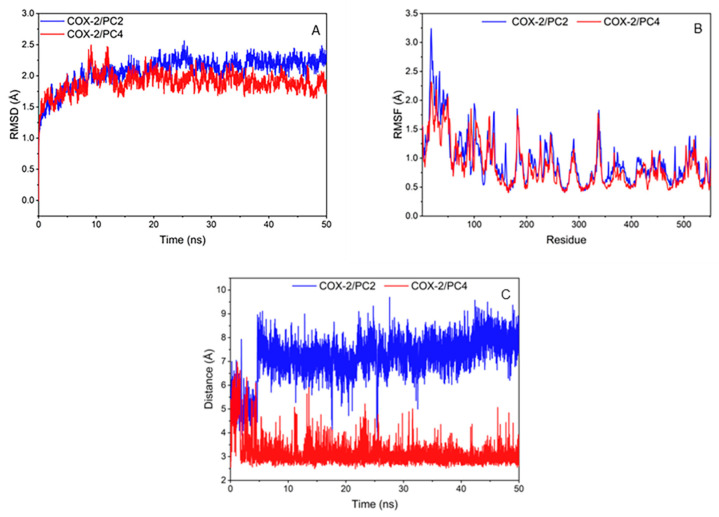
(**A**) Root mean square deviation (RMSD) between amino acid skeleton atoms from cyclooxygenase-2 (COX-2) during simulation; (**B**) Hydrophobic binding diagram of ligand-protein complex and fluctuation of root mean square fluctuation value (RMSF) of amino acid skeleton atoms; (**C**) Distance plot of atom pairs involved in the formation of hydrogen bonds between 7´ oxygen atom of hydroxy-2,4,7-trimethoxyphenanthrene (PC2)/6,7-dihydroxy-2,4-dim ethoxy-phenanthrene (PC4) and hydroxyl of Tyr385 in the course of molecular dynamics simulation trajectory.

The RMSD of compounds and COX-2 amino acid skeleton atom within 50 ns were calculated to test the stability of the system (Figure 4A). The RMSD values of PC2 and PC4 systems basically remained unchanged and reached equilibrium after 20 ns. It is generally considered that the method is feasible when the RMSD value is in the range of 0.1–0.3 nm [27]. The RMSD values of the two complexes in this research were about 0.2 nm, which represented that the simulation results could be used for further analysis.

The root mean square fluctuation value (RMSF) of compounds and COX-2 amino acid skeleton atom were calculated to analyze the fluctuation of the system (Figure 4B). On the whole, the amino acid fluctuation trends of the two systems were roughly similar. PC2 system had low fluctuation at amino acids of 345, 349, 527 and 534, and PC4 system was at amino acids of 349, 385, 527 and 534. The amino acids with low RMSF values in the two systems were consistent with the key amino acids. Hydrogen bond or hydrophobic interaction between the compounds and the amino acids around the COX-2 enzyme binding pocket made the key amino acids more stable, which was conducive to the interaction between ligands and receptors.

In order to further investigate the difference of binding affinity between PC2/PC4 and COX-2 receptor, the binding free energy of the complexes were calculated by MM/PBSA method. Van der Waals energy and electrostatic energy were the most favorable for the binding of the two compounds with COX-2; non-polar solvation was also favorable for binding while, compared with the values of van der Waals energy and electrostatic energy, the contribution of non-polar solvation was weak and the polar solvation was detrimental to the combination of the system (Table 1B). The polar interaction in the solvent largely offset electrostatic energy and as a result the non-polar interaction, especially the dominant van der Waals energy was more conducive to the binding of ligand and receptor. The binding electrostatic energy between PC4 and COX-2 was lower than that of PC2, which might cause the difference in the binding free energy between two polyphenols and protein. As is known, Gibbs free energy must be negative for the reaction to proceed. The values of ΔG_bind_ (binding free energy/Gibbs free energy) for both two phenanthrenes/COX-2 complexes were negative, i.e., reactions between these two phenanthrenes and COX-2 could proceed. On this basis, molecular docking and molecular dynamics simulation were meaningful. A smaller binding free energy value represented lower energy requirement, which profited the binding of ligand and protein. Consequently, it could be concluded that the binding strength of protein-ligand for PC4 was stronger than PC2, coinciding with the molecular docking scoring results.

During MD simulation, it was observed that the hydrogen bond between PC2/PC4 and Tyr385 remained relatively stable after 5 ns (Figure 4C). It was worth noting that the hydroxyl group of Tyr385 was close to the hydroxyl substituent of PC4 (distance around 2 Å), which indicated that PC4 was firmly controlled on the hydroxyl group of Tyr385 in COX-2 through a strong hydrogen bond. Meshram et al. also predicted the existence of strong hydrogen bonds between flavonoids and COX-2 by molecular dynamics simulation [27], indicating that hydrogen bonds played an important role in the combination of natural substances and COX-2. Nevertheless, the distance between the methoxyl group of PC2 and the hydroxyl group of Tyr385 in COX-2 (greater than 6 Å) showed a highly unstable polar interaction between PC2 and Tyr385. Therefore, the binding of PC2 and COX-2 was mainly through van der Waals force and hydrophobic interaction without hydrogen bond, which was consistent with the results of the molecular docking interaction analysis.

To summarize, COX-2 was a good potential target for PC2 and PC4. Due to the hydrogen bond between the hydroxyl group of PC4 (position 7) and Tyr385 site of COX-2 protein, the binding between PC4 and COX-2 protein was stronger than PC2, resulting in higher anti-inflammatory activity.

### 3.3. COX-2 Gene Silencing

PC4 was selected as the research object owing to its stronger anti-inflammatory activity and COX-2 molecular binding ability. To further confirm whether COX-2 was the target of Chinese yam active ingredient regulating NF-κB/COX-2 pathway, the effects of PC4 on the protein expression of the main inflammatory pathway were evaluated by western blot in COX-2 gene silenced cells.

**Figure 5:**
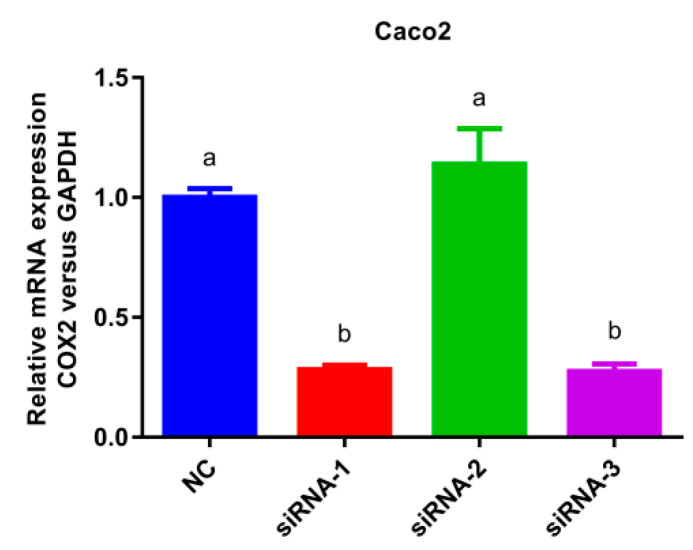
Histogram of mRNA expression after cyclooxygenase-2 (COX-2) silencing. Values without a common letter were significantly different as *p* < 0.05. Glyceraldehyde-3-phosphate dehydrogenase (GAPDH) was set as loading control, normal cells group (NC) was set as control group.

The COX-2 gene silencing Caco-2 cell model was established as presented in Figure 5. In comparison with the normal cells group, the mRNA expression of siRNA-1 and siRNA-3 decreased significantly (*p* < 0.05) without significant differences between them, indicating that siRNA-1 and siRNA-3 exhibited good COX-2 gene silencing effect. However, there was no significant change in the expression of siRNA-2 compared to NC group, thus siRNA-1 or siRNA-3 was selected as the silencing plasmid.

**Figure 6:**
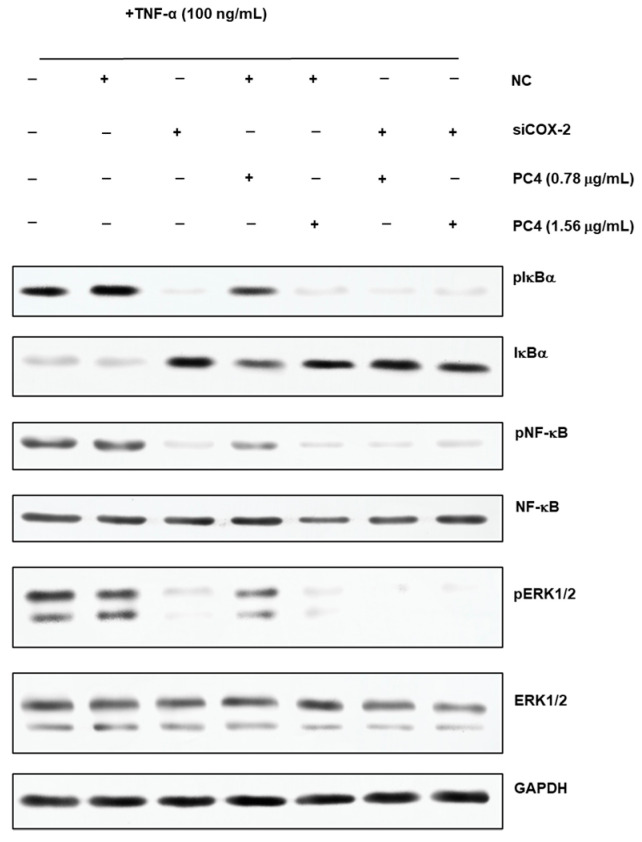
Western blot images of phosphorylation nuclear factor kappa-B inhibitor alpha (pIkBα), phosphorylation nuclear factor kappa-B (pNF-κB), phosphorylation extracellular signal-regulated kinase 1/2 (pERK1/2), IkBα, NF-κB, ERK1/2 and glyceraldehyde-3-phosphate dehydrogenase (GAPDH). +means added, and −means not added. NC means normal cell group, PC2 means hydroxy-2,4,7-trimethoxyphenanthrene and PC4 means 6,7-dihydroxy-2,4-dim ethoxy-phenanthrene.

**Figure 7:**
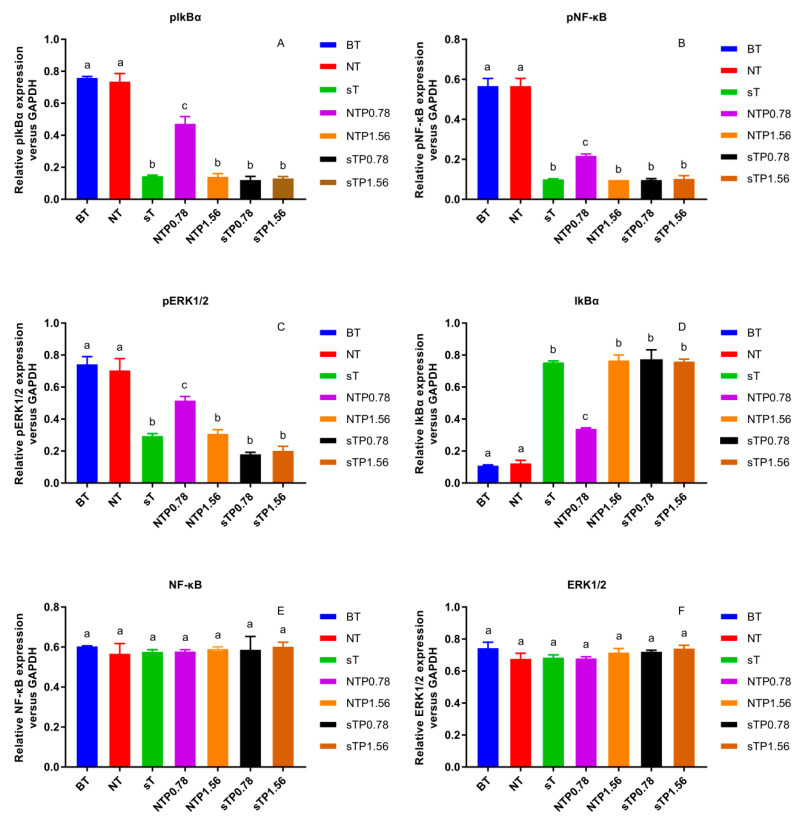
Effect of CP4 on relative protein expression in normal and cyclooxygenase-2 (COX-2) silencing human colon adenocarcinoma (Caco-2) cells (**A**) Relative expression of phosphorylation nuclear factor kappa-B inhibitor alpha (pIkBα), (**B**) Relative expression of phosphorylation nuclear factor kappa-B (pNF-κB), (**C**) Relative expression of phosphorylation extracellular signal-regulated kinase 1/2 (pERK1/2), (**D**) Relative expression of IkBα, (**E**) Relative expression of NF-κB, (**F**) Relative expression of ERK1/2. Blank group with TNF-α (BT), normal cell group with TNF-α (NT), COX-2 silenced cell group with TNF-α (sT), normal cell group with TNF-α and 0.78 μg/mL drug interven-tion (NTP0.78), normal cell group with TNF-α and 1.56 μg/mL drug intervention (NTP1.56), COX-2 silenced cell group with TNF-α and 0.78 μg/mL drug intervention (sTP0.78), COX-2 silenced cell group with TNF-α and 1.56 μg/mL drug intervention (sTP1.56) were treated with drugs. Values without a common letter were significantly different as *p* < 0.05.

Our previous research reported that the NF-κB/COX-2 signaling pathway played an important role in the anti-inflammatory process of PC4 [22]. Hence, the changes in protein level on NF-κB/COX-2 signaling pathway were detected in normal and COX-2 gene silencing Caco-2 cells (Figure 6) and the results were concluded in Figure 7. There was no significant difference in target protein level between BT group and NT group, indicating that reagents in the transfection process basically did not affect the expression of the target gene, indicating the reliability of the subsequent analysis. PC4 could inhibit pIkBα and pNF-κB with a dose-dependent manner in NTP groups without COX-2 gene silencing. However, compared with sT group, there was no significant difference in the expression of pIkBα and pNF-κB protein among sT, sTP0.78 and sTP1.56 groups after COX-2 gene silencing, which indicated that PC4 did not directly act on IkBα and NF-κB protein in the pathway, while inhibiting inflammation by binding with COX-2. This result further confirmed that COX-2 was supposed to be the target of PC4 in the NF-κB/COX-2 pathway.

ERK1/2 protein is a kinase located upstream of NF-κB protein that can activate NF-κB transcription protein [17]. As shown in Figure 7C, PC4 pretreatment significantly inhibited pERK1/2 protein level with a dose-dependent manner in NTP groups without COX-2 gene silencing. However, the expression of pERK1/2 protein in sTP0.78 and sTP1.56 groups had no significant changes compared with sT group after COX-2 gene silencing, suggesting that PC4 did not inhibit ERK1/2 protein in MAPK pathway.

In this experiment, pIkBα, pNF-κB and pERK1/2 protein levels in sT group were similar to that in NTP1.56 group, which indicated that the lack of COX-2 enzyme could inhibit the inflammation of Caco-2 cells, and further confirmed the key role of COX-2 enzyme in inflammation response. Studies have shown that chronic inflammation induced COX-2 expression and COX-2, PGE2, EP2 and NF-κB produced positive feedback loops to amplify inflammatory signals [18,28], which might explain why PC4 could still influence the expression of its upstream proteins IkBα, NF-κB and ERK1/2 after acting on the downstream inflammatory factor COX-2 enzyme.

In addition, there was no significant difference in NF-κB and ERK1/2 protein levels among the treatment groups, while IkBα and pIkBα had the opposite expression trends. The inflammatory pathway mainly functions through phosphorylated proteins. The change trends of pIkBα, pNF-κB and pERK1/2 in different groups were roughly the same, which was consistent with the results of Guo and Shen et al. [29,30]. The reason for the same trend was that NF-κB dimer binds to IkBα in an inactive state, then IkBα phosphorylates to form pIkBα and dissociates from NF-κB when cells were stimulated; ultimately, NF-κB enters the nucleus to start the downstream inflammatory pathway. Simultaneously, ERK1/2 is the upstream protein of NF-κB which can regulate the activation of NF-κB protein. Therefore, the expression of pNF-κB protein was consistent with pERK1/2. In conclusion, PC4 was a good inhibitor of COX-2 enzyme, which exerted anti-inflammatory activity by inhibiting COX-2 protease and regulated the expression of pIkBα, pNF-κB and pERK1/2 proteins through a positive feedback loop.

## 4. Discussion

The global incidence rate of ulcerative colitis is increasing year by year and its pathogeny includes intestinal mucosal injury, environmental trigger and intestinal microbial effects, etc. [31,32], From our previous report, ulcerative colitis mice models were established to study the intestinal mucosal protective effect and mechanism of effective components in Chinese yam [22]. The cell polarity and tight junction of human colon adenocarcinoma cells (Caco-2 cells) are similar to small intestinal epithelial cells, which can be used to effectively study the expression of inflammatory factors [33]. Janus kinase-signal transducer and activator of transcription (JAK-STAT), mitogen-activated protein kinase (MAPK) and nuclear factor kappa-B (NF-κB) signaling pathways are three important inflammatory signaling pathways in cells [34]. Besides, tumor necrosis factor-α (TNF-α) can activate extracellular signal-regulated kinase 1/2 (ERK1/2) and NF-κB through tumor necrosis factor receptor 1 to promote inflammation, while research on TNF-α directly activating JAK-STAT pathway has not been reported. Based on the preliminary anti-inflammatory mechanism of 6,7-dihydroxy-2,4-dimethoxyphenan- threne (PC4), the prediction and confirmation of inflammatory targets were focused on NF-κB/cyclooxygenase-2 (COX-2) pathway.

Tumor necrosis factor-α (TNF-α) and interleukin-8 (IL-8) are the most common inflammatory factors [25] and our results showed that the levels of TNF-α and IL-8 in inflammatory Caco-2 cells model group were higher than those in the control group, indicating that TNF-α successfully induced cellular inflammatory response. TNF-α is considered to aggravate the severity of inflammatory response and damage to gastrointestinal tissue and mucosa [35], which has remarkable impact on the prognosis. Moreover, IL-8 can activate neutrophils and promote their migration into the blood [36]. Its content is positively correlated with the severity of inflammation, which can be used as a reliable index to evaluate the illness degree of ulcerative colitis.

From our previous research, phenanthrene compounds 1~4 exhibited higher COX-2 inhibition than positive control aspirin, ibuprofen and naproxen. This research further verified the binding of PC4 with COX-2 by molecular docking and molecular dynamics simulation. Molecular docking technology possesses apparent advantages in active ingredients-pharmacological targets research and has been widely used in the discovery, development and efficacy mechanism of active ingredients. Compared with 6-hydroxy-2,4,7-trimethoxyphenanthrene (PC2), PC4 had stronger interaction with COX-2 due to the formation of a hydrogen bond at Tyr385 residue. Interestingly, it has been experimentally validated that the phenolic oxygen of Tyr385 plays a crucial role in abstracting hydrogen from eicosapentaenoic acid (EPA) and arachidonate substrates [37]. Therefore, Tyr385 is considered as a well-established catalytic site in COX reactions [38]. The molecular docking results of Meshram et al. also showed that Tyr385 was a crucial hydrogen bond binding site between a variety of small bioactive substances and COX-2 [27]. Therefore, combined with the molecular dynamics simulation analysis of this study, it was proved that Tyr385 played an important role in PC4-mediated COX-2 inhibition. Residues of Val349, Ala527 and Leu534 were the hydrophobic binding sites of PC4 and COX-2 protein, which exhibited non-negligible effects.

Under pathological conditions, the gene expression of COX-2 increases several times, thus responding to proinflammatory factors quickly. Simultaneously, gene silencing is considered to be an effective method to screen drug targets. COX-2 was predicted as the target of PC4 through molecular docking results in this experiment. To further confirm this conclusion, protein changes in NF-κB/COX-2 pathway were analyzed after silencing COX-2 gene in Caco-2 cells.

NF-κB plays a crucial role in immune and inflammatory response. Nuclear factor kappa-B inhibitor alpha (IκBα) is known as an inactive inhibitor protein of NF-κB p50/NF-κB p65 heterodimer and the combination of IκBα and dimer is localized in the cytoplasm. After degradation of inhibitor protein, the activated dimer enters the nucleus and immediately starts the transcription of downstream inflammatory factors such as TNF-α, IL-8 and COX-2 [39]. In recent years, multiple natural compounds that can inhibit NF-κB pathway have been reported to control the health threat of NF-κB in organisms. The main pathways by which they inhibit NF-κB are as follows: (1) reducing the formation of NF-κB activator as antioxidants; (2) inhibiting IκBα phosphorylation and degradation; (3) reducing protease activity, thereby inhibiting Rel/NF-κB [40,41]. Yuan et al. reported the anti-inflammatory mechanism of *Salvia miltiorrhiza* Bunge on NF-κB pathway, which indicated that dihydrotanshinone blocked the dimerization of Toll-like receptors 4 to form myeloid differentiation primary response 88 and activated TGF-β-activated kinase 1 thereby inhibiting NF-κB and MAPK pathways [42]. As a phytochemical compound, PC4 can inhibit NF-κB/COX-2 pathway, while the mechanism by which PC4 regulates NF-κB is still unclear so far.

**Figure 8:**
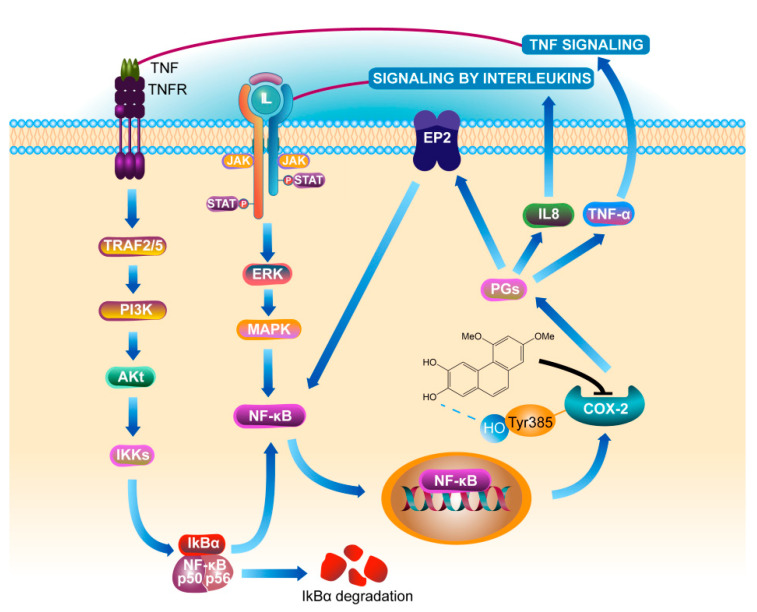
Mechanism of PC4 treating ulcerative colitis by inhibiting COX-2. Definitions of the abbreviations in this figure are below: tumor necrosis factor receptor (TNFR), tumor necrosis factor receptor-associated factors (TRAF), phosphatidylinositol-3-kinase (PI3K), protein kinase B alpha (namely Akt), nuclear factor kappa-B inhibitor alpha (IkBα), nuclear factor kappa-B (NF-κB), interleukin 8 (IL8), Janus kinase (JAK), signal transducer and activator of transcription (STAT), extracellular signal-regulated kinase (ERK), mitogen-activated protein kinase (MAPK), cyclooxygenase-2 (COX-2), prostaglandins (PGs), E-series of prostaglandin receptors type 2 (EP2), tumor necrosis factor-α (TNF-α).

Phosphorylated protein can better reflect the activity of protein in cells. This study found that there was no significant change in the expression levels of pIkBα and pNF-κB protein in COX-2 gene silenced cells regardless of whether PC4 was added or not, indicating that PC4 as COX-2 enzyme inhibitor did not act on the IkBα and NF-κB targets directly. COX-2 is the key enzyme which inhibits the formation of PGs. Meanwhile, ulcerative colitis is a chronic nonspecific inflammation. Studies have confirmed that prostaglandins (PGs) existed in the positive feedback loop of chronic diseases [18], which generated a positive feedback loop through COX-2, prostaglandin E2, E-series of prostaglandin receptors type 2 (EP2) and NF-κB to amplify inflammatory signals [18,28]. This explained the phenomenon that the protein levels of pIkBα, pNF-κB and pERK1/2 in the upstream were also changed after PC4 acted on COX-2 enzyme. Aoki et al. [43] observed that the occurrence of intracranial aneurysm (IA) was induced by COX-2 in the IA model. They further found that EP2 was up-regulated at the IA site and the signal pathway was inhibited of in mice with IA lacking EP2. Activated NF-κB induced various inflammation related genes, hence inhibition of COX-2 to block EP2 expression and knockout of EP2 to inhibit COX-2 induction in IA, both inhibited NF-κB activation and then inhibited COX-2 expression. This suggested that the inflammatory therapeutic value of PC4 contributed to its inhibition of the positive feedback loop of NF-κB/COX-2. In addition, PGs catalyzed by COX-2 enzyme could regulate the expression of IL-8 [44]. TNF-α and IL-8 are not only NF-κB inflammatory pathway inducers, but also downstream effectors. This indicated that PC4 might down-regulate the expression of TNF-α and IL-8 after inhibiting the COX-2 enzyme, thereby reducing the initial inducers of inflammatory pathway and finally inhibiting the whole inflammatory process. The anti-inflammatory mechanism of PC4 on NF-κB/COX-2 pathway is summarized in Figure 8.

As a member of MAPK pathway, ERK 1/2 is the upstream kinase of NF-κB protein [17], which plays an important role in regulating the stress inflammatory response, proliferation and differentiation of gastric mucosal epithelium. Phosphorylated ERK can transmit extracellular signals to the nucleus. The study by Kundu et al. showed that COX-2 enzyme was closely related to MAPK pathway and green tea polyphenols inhibited the catalytic activity of ERK, suggesting that ERK may be a potential target of polyphenol antitumor activity [45]. The level of pERK 1/2 was also analyzed in this experiment. However, it was found that the target of PC4 was not pERK1/2, but the downstream COX-2 protease, which was different from the conclusion of Kundu et al.

In general, from the perspective of molecular groups, the phenolic oxygen of Tyr-385 had been experimentally validated to play a vital role of abstracting the 13-proS hydrogen from EPA and arachidonate substrates [37]. That is to say, when PC4 and Tyr385 on COX-2 were combined by hydrogen bonds, it might lead to the reduction of the sites on COX-2 enzyme that catalyze arachidonate substrates, thereby reducing the production of downstream inflammatory factor PGs and inhibiting inflammatory reaction. This provided molecular basis for COX-2 as a potential target for PC4 to alleviate inflammation. In addition, the actual combination of PC4 and COX-2 were also reflected in the in vitro experiments we had studied previously. The anti-inflammatory activity of PC2 and PC4 had been verified by the COX-2 enzyme inhibitory activity in vitro [21] and the cell activity experiment in this experiment. Our preliminary work had confirmed that PC4 alleviated DSS-induced intestinal mucosal injury in mice via modulation of the NF-κB/COX-2 pathway [22]. Combining the results of molecular binding, actual binding and gene knockout, it is suggested that COX-2 was one of the targets of PC4 in alleviating ulcerative colitis.

However, this study also has certain limitations. Firstly, the binding of bioactive compounds and protein targets was investigated only by a simulated method, which might not reflect their actual binding enough. Secondly, only the prediction and verification of the target of PC4 was carried out in the present research. However, PC4 might partly be digested and metabolized after entering the digestive tract and would participate in the metabolism of inflammatory pathways in the form of its metabolites. Therefore, isothermal titration calorimetry, drug affinity responsive target stability, microscalethermophoresis and surface plasmon resonance will be used to further confirm the actual binding of PC4 and the metabolites of PC4 in vivo and the mechanism in alleviating colitis will be further explored in the next step of our research.

## 5. Conclusions

In summary, Chinese yam polyphenol PC4 could relieve intestinal inflammation as COX-2 inhibitor. Among the two active components of Chinese yam polyphenols, PC4 exhibited better anti-inflammatory activity and stronger molecular binding to COX-2, which was due to the role of the hydrogen bond between 7´ hydroxyl group of PC4 and Tyr385 residue of COX-2 protein. In addition, PC4 inhibited the protein expression of NF-κB/COX-2 pathway in TNF-α-induced Caco-2 cells, but no significant difference had been observed for the pathway protein in COX-2 gene silenced cells. PC4 might realize its anti-inflammatory effect in Caco-2 cells by inhibiting COX-2 protease and inhibit the amplification of the initial inflammatory signal by the positive feedback loop to enhance the anti-inflammatory activity. This study revealed the potential target and mechanism of Chinese yam polyphenol PC4 regulating NF-κB/COX-2 pathway, which provided a theoretical basis and reference for Chinese yam to protect intestinal mucosal injury and exert the effect of “strengthening intestines and stomach”.

## Figures and Tables

**Table 1:** (**A**) Docking scores and interaction analysis of hydroxy-2,4,7-trimethoxyphenanthrene (PC2), 6,7-dihydroxy-2,4- dimethoxy-phenanthrene (PC4) docking with cyclooxygenase-2 (COX-2). (**B**) Binding energy of two compounds with COX-2 protein (Note. ΔE_vdw_: van der Waals energy; ΔE_elec_: electrostatic energy; ΔG_GB_: electrostatic contribution to solvation; ΔG_SA_: non-polar contribution to solvation; ΔG_bind_ (=G (receptor and ligand complex) − G (receptor) − G (ligand)): binding free energy, is also called Gibbs free energy).

(A)			
Compounds	G-Score/(kJ·mol^−1^)	Hydrogen Binding	Hydrophobic Interaction
PC4	−8.109	Tyr385	Val349, Ala527, Leu534
PC2	−7.997	/	Ile345, Val349, Ala527, Leu534
**(B)**			
**System Name**		**COX-2/PC2**	**COX-2/PC4**
ΔE_vdw_ (van der Waals energy)		−39.88 ± 1.16	−38.46 ± 2.18
ΔE_elec_ (electrostatic energy)		−5.47 ± 2.22	−9.73 ± 2.03
ΔG_GB_ (electrostatic contribution to solvation)		21.05 ± 1.71	21.37 ± 1.10
ΔG_SA_ (non-polar contribution to solvation)		−5.34 ± 0.07	−5.31 ± 0.11
ΔG_bind_ (binding free energy/Gibbs free energy/)		−29.65 ± 1.77	−32.14 ± 1.94

## Data Availability

The data presented in this study are available on request from the corresponding author.

## Abbreviations

COX-2, cyclooxygenase-2; NF-κB, nuclear factor kappa-B; IL-8, interleukin-8; TNF-α, tumor necrosis factor-α; PC2, 6-hydroxy-2,4,7-trimethoxyphenanthrene; PC4, 6,7-dihydroxy-2,4-dimetho xyphenanthrene; Caco-2, human colon adenocarcinoma; pIkBα, phosphorylation nuclear factor kappa-B inhibitor alpha; pNF-κB, phosphorylation NF-κB; pERK1/2, phosphorylation extracellular signal-regulated kinase 1/2; MAPK, mitogen-activated protein kinase; PGs, prostaglandins; PGE2, prostaglandin E2; EP2, E-series of prostaglandin receptors type 2; DSS, dextran sulfate sodium salt; ATCC, American model culture collection; NPT, isobaric-isothermal; RMSD, root mean square deviation; MM/PBSA, molecular mechanics generalized Born surface area; NC, normal Caco-2 cells; Sh, COX-2 silenced Caco-2 cells; BT, blank group with TNF-α; NT, normal cell group with TNF-α; sT, COX-2 silenced cell group with TNF-α; NTP0.78, normal cell group with TNF-α and 0.78 μg/mL drug intervention; NTP1.56, normal cell group with TNF-α and 1.56 μg/mL drug intervention; sTP0.78, COX-2 silenced cell group with TNF-α and 0.78 μg/mL drug intervention, sTP1.56, COX-2 silenced cell group with TNF-α and 1.56 μg/mL drug intervention; ANOVA, analysis of variance; RMSF, root mean square fluctuation value; JAK-STAT, Janus kinase-signal transducer and activator of transcription; EPA, eicosapentaenoic acid; IA, intracranial aneurysm; TNFR, tumor necrosis factor receptor; TRAF, tumor necrosis factor receptor-associated factors, PI3K, phosphatidylinositol-3-kinase.
